# 小细胞肺癌卵巢转移2例及文献回顾

**DOI:** 10.3779/j.issn.1009-3419.2010.12.16

**Published:** 2010-12-20

**Authors:** 成英 孙, 晓岚 刘, 国云 张, 伟力 王

**Affiliations:** 110032 沈阳，中国医科大学附属第四医院放疗科 Department of Radiation, the Fouth Afiliated Hospital of China Medical University, Shenyang 110032, China

小细胞肺癌（small cell lung cancer, SCLC）是恶性度极高的肺部肿瘤之一，它具有广泛的转移倾向，极易发生血行转移，尤以肝、脑、骨转移常见^[1]^，而卵巢转移罕见。下面就我院2年内2例SCLC卵巢转移患者的临床特点进行讨论。

## 1 临床资料

患者1，女性，54岁，2007年3月因咳嗽行肺CT检查发现右肺肿物，经纤维支气管镜检查示右肺中间干支气管管口菜花样肿物，活检病理确诊小细胞肺癌（图 1A），行EP方案（顺铂120 mg，d1；足叶乙甙100 mg，d1-d3）化疗6周期，第2周期结束时肺部CT检查示病灶消失（图 1B），第3周期-第4周期化疗与放疗同步，剂量50 Gy/27 f。2008年4月-5月行全脑预防放疗，剂量30 Gy/16 f。2008年11月PET-CT检查示盆腔局部代谢增高（图 1C），彩超示左侧卵巢占位性病变，CA125 15.3 U/mL（正常值为0 U/mL-35 U/mL），2008年11月行全子宫+双附件切除+盆腔淋巴结清扫术，术后病理（图 2）：左侧附件球形结节，大小15 cm×12 cm×8.5 cm，包膜完整。诊断为左卵巢神经内分泌癌（雀麦细胞型），腹膜结节性转移。术后行CTX+CBP方案（环磷酰胺0.8 mg，d1；卡铂500 mg，d1）化疗6周期。2009年9月发现腹膜后淋巴结转移，EP方案（剂量同前）化疗4个周期并同步给予腹膜后转移淋巴结及左侧盆腔淋巴引流区放疗，剂量25.5 Gy/16 f。2010年1月因腹部病变进展给予IP周方案（伊立替康80 mg，d1、d8、d15；顺铂50 mg，d1-d2）化疗1周，因严重骨髓抑制而中止，1月末头部增强MRI示右侧小脑及左额叶结节影，伴周围水肿，再次予放疗，全脑30 Gy/15 f，右侧小脑转移灶40 Gy/20 f，同步行TP方案（紫杉醇180 mg，d1；顺铂40 mg，d1-d3）化疗1周期。3月末因左锁骨上淋巴结肿大伴声音嘶哑，行左锁骨上淋巴结区放疗，剂量25 Gy/10 f，放疗后肿物消失，声音嘶哑缓解，因骨髓抑制明显（WBC < 3.0×10^9^）未行全身化疗。2010年6月入院复查发现左侧肾脏后方软组织影，腹主动脉旁软组织影，神经元特异性烯醇化酶（neuron specific enolase, NSE）升至144 ng/mL，拟给予TAX（紫杉醇）30 mg，周方案化疗及局部放疗。

**1:**
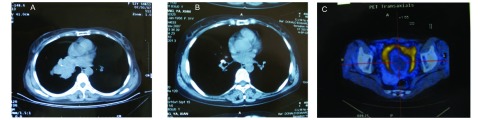
患者1的影像学特点。A：确诊时肺CT；B：化疗2周期后肺CT；C：PET-CT示腹腔代谢增高。 CT imaging of case one, 54-year-old woman with SCLC presented with a left ovarian metastases. A: lung imaging on CT when diag- nosed; B: lung imaging on CT after two circles of chemotherapy; C: pelvic cavity imaging on PET.

**2:**
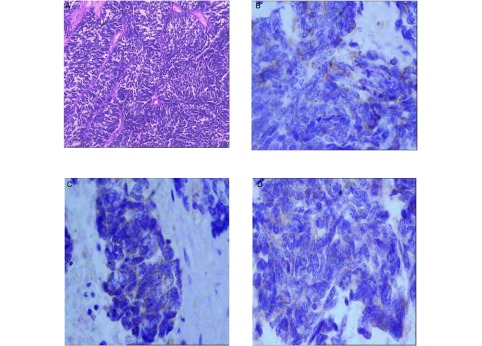
患者1卵巢转移小细胞肺癌肿瘤细胞（HE, ×100）及不同免疫组化指标中细胞器结构的阳性表现（SP, ×400）。A：肺癌肿瘤细胞；B：CgA；C：NSE；D：Syn。 Case one: the organoid of SCLC metastatic to the ovary (HE, ×100) and positive expression of SCLC metastatic to the ovary are showing for various immunohistochemical marker (SP, ×400). A: lung cancer tumor cell; B: CgA; C: NSE; D: Syn.

患者2，女性，34岁，2009年10月末因胸闷、气短行胸部CT示右肺中叶占位，纤维支气管镜检查示右肺中叶管口菜花样肿物，表面坏死，活检病理确诊（右肺）小细胞肺癌（中间型），行EP方案（顺铂25 mg/m^2^，d1-d3；足叶乙甙100 mg/m^2^，d1-d3）化疗2个周期，复查肺CT示肿物缩小不明显，改为IEP方案（异环磷酰胺1 200 mg/m^2^ d1-d3；足叶乙甙75 mg/m^2^，d1-d3；顺铂20 mg/m^2^，d1-d4）化疗2周期，化疗后病灶缩小仍不明显，给予TP方案（艾素120 mg，d1；顺铂20 mg，d1-d4）化疗1个周期后（图 3A），于2010年3月-4月行右肺原发灶、纵隔淋巴引流区及双锁骨上区放疗，右肺原发灶、纵隔淋巴引流区剂量为42 Gy/21 f，双锁骨上区剂量为46 Gy/23 f，放疗中因发现多发骨转移及盆腔肿物而中止（图 3C）。CA125为35.6 U/mL。2010年5月行盆腔肿瘤细胞减灭术，术后病理报告为：双侧卵巢小圆细胞恶性肿瘤，符合SCLC双侧卵巢转移癌（图 4），放疗后2个月复查肺CT示肺内病灶明显缩小（图 3B）。

**3:**
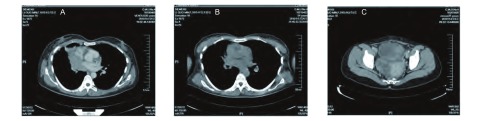
患者2的影像学特点。A：化疗5周期后（即放疗前）肺CT；B：放疗后2月肺CT；C：盆腔CT。 CT imaging of case two, 34-year-old woman with SCCL presented with bilateral ovarian metastases; A: lung imaging on CT after five circles of chemotherapy; B: lung imaging on CT after two months of radiotherapy; C: pelvic cavity imaging on CT.

**4:**
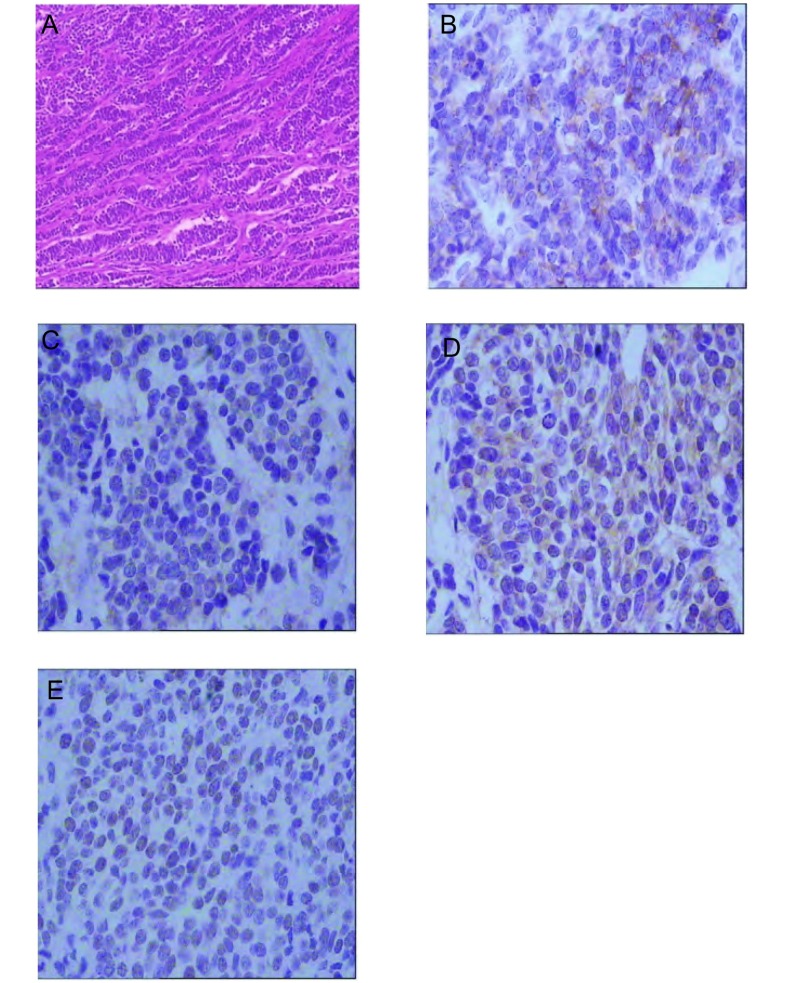
患者2卵巢转移小细胞肺癌肿瘤细胞（HE, × 100）及不同免疫组化指标中。肿瘤细胞的阳性表现（SP, ×400）。A：肺癌肿瘤细胞；B：CgA；C：NSE；D：CD99；E：TTF-1。 Case two: the organoid of SCLC metastatic to the ovary (HE, ×100) and positive expression of SCLC metastatic to the ovary are showing for various immunohistochemical marker(SP, ×400). A: lung cancer tumor cell; B: CgA; C: NSE; D: CD99; E: TTF-1.

## 2 讨论

国内外文献^[1]^报道卵巢转移癌占卵巢恶性肿瘤的6%-28%，其中以胃癌最常见，占60%-80%^[2, 3]^。SCLC发生卵巢转移临床罕见。国内仅中科院肿瘤医院报告23年中发现6例，发病率仅1.2%^[4]^。国外文献^[5-7]^也多为小样本病例分析。复习国内外相关文献，总结SCLC卵巢转移特点如下：

### 2.1 临床特点

① 发病年龄不等，26岁-85岁，但多见于绝经前。主要原因可能为:绝经前生殖期卵巢血运丰富、功能旺盛，为肿瘤转移及生长提供了有利条件；②大部分患者先出现肺部症状，后出现腹部症状，以腹胀、阴道流血多见，多在确诊SCLC后1年左右^[5, 6]^出现，以卵巢转移为首发症状的小细胞肺癌少见；③临床分期多为弥漫性病变（6/8），肝转移少见。本院2例及中科院肿瘤医院6例患者均无肝转移。诊断卵巢转移时疾病多进展；④CA125水平低。研究显示：大约80%原发卵巢癌CA125 > 65 U/mL^[8]^，平均630 U/mL^[9]^，而转移性卵巢癌仅33%患者CA125 > 65 U/mL，很少高于500 U/mL^[8]^。本院2例患者CA125均 < 65 U/mL；⑤影像学特点：盆腔内子宫体前方囊实性病变，内见不规则液性低密度影，增强可见强化。

### 2.2 病理特点

① 大体标本：单侧或双侧均见，直径较大，多在10 cm左右，为多发囊实性包块，包膜多完整。切面灰白或灰黄，质脆易碎；②显微镜：瘤细胞较小，类圆形或不规则形，染色质细腻，胞浆少或无，无核仁，核分裂相易见。细胞坏死多见，广泛淋巴血管侵袭，但侵及卵巢表面间皮细胞少见；③免疫组化：NSE、嗜铬素A（chromogranin A, CgA）、突触素（synaptophysin, SYN）是SCLC的较特异指标，Rund等^[10]^研究显示：核周的、点状细胞角蛋白20（cytokeratin 20, CK20）染色有助于区分卵巢原发小细胞癌及SCLC转移所致；④基因检测：Garcia等^[11]^检测1例SCLC卵巢转移患者肺原发灶与卵巢转移灶*p53*基因突变情况，发现两者具有相同的突变位点（S215）。

### 2.3 诊治及评估预后

SCLC是一种恶性度很高的恶性肿瘤，一般经过综合治疗后，局限期患者的中位生存期为12个月-16个月，5年生存率为10%-15%；而广泛期患者的中位生存期仅7个月-10个月，生存2年者少见，5年生存率为0^[12]^。国内资料^[4]^显示虽然对伴有卵巢转移的SCLC进行了积极的治疗，其中位生存期只有17个月，但发生卵巢转移后中位生存时间则缩短为8个月，说明卵巢转移预后很差。即使如此，SCLC发生卵巢转移后如患者身体状态允许，建议行卵巢转移癌根治术或减瘤术，为后续放化疗创造条件。患者1卵巢转移癌术后虽然出现多处转移，但给予积极化放疗后，目前已存活22个月。
